# The host mannose-6-phosphate pathway and viral infection

**DOI:** 10.3389/fcimb.2024.1349221

**Published:** 2024-01-31

**Authors:** Qincheng Liu, Weiqi Wang, Liwei Xu, Qisheng Zhang, Hongna Wang

**Affiliations:** ^1^ Affiliated Cancer Hospital & Institute of Guangzhou Medical University, Guangzhou, China; ^2^ Key Laboratory for Cell Homeostasis and Cancer Research of Guangdong Higher Education Institutes, Guangzhou, China; ^3^ Shanghai Sino Organoid Lifesciences Co., Ltd., Shanghai, China

**Keywords:** virus, cathepsin, M6P, LYSET, GNPT, M6PR, lysosome, infectious diseases

## Abstract

Viruses, despite their simple structural composition, engage in intricate and complex interactions with their hosts due to their parasitic nature. A notable demonstration of viral behavior lies in their exploitation of lysosomes, specialized organelles responsible for the breakdown of biomolecules and clearance of foreign substances, to bolster their own replication. The man-nose-6-phosphate (M6P) pathway, crucial for facilitating the proper transport of hydrolases into lysosomes and promoting lysosome maturation, is frequently exploited for viral manipulation in support of replication. Recently, the discovery of lysosomal enzyme trafficking factor (LYSET) as a pivotal regulator within the lysosomal M6P pathway has introduced a fresh perspective on the intricate interplay between viral entry and host factors. This groundbreaking revelation illuminates unexplored dimensions of these interactions. In this review, we endeavor to provide a thorough overview of the M6P pathway and its intricate interplay with viral factors during infection. By consolidating the current understanding in this field, our objective is to establish a valuable reference for the development of antiviral drugs that selectively target the M6P pathway.

## 1 Introduction

As obligate intracellular parasites, viruses seize control of various host cell processes during the infection cycle, actively manipulating or modifying the cellular environment to establish optimal conditions for their replication. Lysosomes, found within eukaryotic cells, are acidic organelles enclosed by a single membrane. They house a diverse array of more than 60 hydrolytic enzymes. Through mechanisms like endocytosis, autophagy, and phagocytosis, lysosomes facilitate the degradation of a wide range of macromolecules and eliminate damaged organelles. Lysosomes originate from the intracellular vesicular transport system. Certain components of lysosomes are derived from extracellular sources and are internalized through endocytosis into endocytic vesicles, which undergo maturation and transform into late endosomes upon fusion with early endosomes. Subsequently, late endosomes receive vesicles loaded with processed hydrolases from the Golgi apparatus and undergo further maturation, ultimately transforming into fully functional lysosomes (Perera and Zoncu, 2016; Lawrence and Zoncu, 2019; Yang and Tan, 2023). During this process, a pH gradient is established, with early endosomes having a pH range of 6.0-6.5, late endosomes having a pH range of 5.5-6.0, and mature lysosomes maintaining a pH of 4-5. This pH gradient, combined with the presence of various types of single-membrane-bound endosomes, creates favorable conditions for viruses to enter the cell (Marsh and Helenius, 2006). Through the mechanism of endocytosis, viruses effectively sequester themselves from the cytoplasm and other cellular constituents. This strategic isolation shields their components from potential damage caused by cytoplasmic proteins and early detection by intracellular antiviral defense systems. Additionally, viruses depend on the sequential utilization of endosomes and lysosomes to navigate from the periphery of the cytoplasm toward regions proximal to the cell nucleus, thereby gradually infiltrating the interior of the cell. The alterations in pH that occur during the transition from endosomes to lysosomes serve as cues for viruses to determine their intracellular localization (Mercer et al., 2010).

## 2 The components of the M6P pathway

Lysosomes contain more than 50 acid hydrolases responsible for breaking down a wide range of substances, including proteins, nucleic acids, polysaccharides, and lipids. These enzymes are specifically designed to function in acidic environments and become inactive in the neutral pH of the cytoplasm. This unique property provides a protective mechanism for the cell, ensuring that even in the event of lysosomal rupture, the released hydrolases do not uncontrollably degrade cellular materials due to the unfavorable neutral pH. Furthermore, these hydrolases are heavily glycosylated, which offers them protection against the extreme acidity within the lysosome (Eskelinen et al., 2003; Zoncu and Perera, 2022).

The biosynthesis of lysosomal hydrolases follows a pattern similar to secretory proteins. Initially, they are synthesized as immature enzyme precursors with signal peptides attached to ribosomes on the endoplasmic reticulum (ER). These signal peptides guide the hydrolases into the ER lumen, where they undergo initial processing. This includes the addition of various sugar molecules such as glucose, mannose, and N-acetylglucosamine (GlcNAc). Among these modifications, *N*-linked glycosylation at the asparagine (Asn) site is the most prevalent, where a high-mannose oligosaccharide chain is attached to an Asn residue of the hydrolases via GlcNAc. Partially matured enzymes are then transported from the ER to the Golgi apparatus via vesicles (Saftig and Klumperman, 2009; Mindell, 2012).

**Figure 1:**
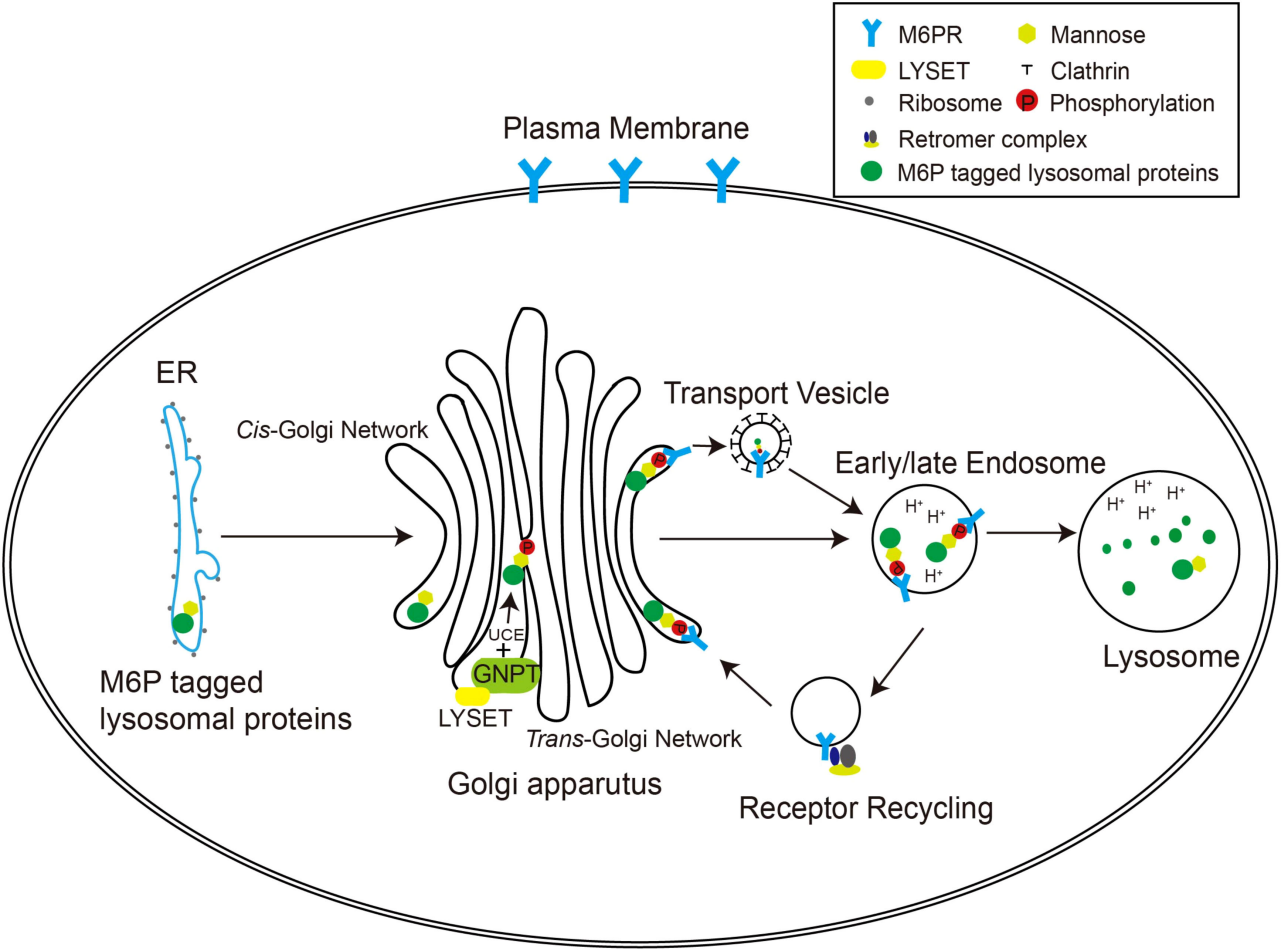
The M6P pathway in mammalian cells. The M6P-tagged lysosomal proteins are synthesized in the rough ER as immature *N*-linked high mannose-type oligosaccharides, which are then transferred to the Golgi apparatus for M6P modification. In the *cis*-Golgi network, the GNPT/LYSET complex and UCE phosphorylate certain mannose residues into M6P sequentially. The M6P groups are then recognized by M6PRs in the TGN. In this way, lysosomal proteins destined for the endosomal–lysosomal system are packaged into clathrin-coated transport vesicles and sorted away from secretory proteins. The acidic environment of the endosome separates M6PRs from the cargoes, and M6PRs are recycled back to the Golgi apparatus via the retromer complex.

In higher organisms, the mannose 6-phosphate (M6P) pathway has evolved to ensure the specific delivery of hydrolases to lysosomes, rather than other cellular compartments (**Figure 1**). This pathway allows the hydrolases to acquire a distinctive M6P marker, ensuring their proper transport to lysosomes for effective functionality. The formation of the M6P marker primarily occurs within the Golgi apparatus. In the *cis* Golgi cisternae, the GlcNAc-1-phosphotransferase (GNPT) of the M6P pathway recognizes the hydrolases and adds M6P markers to selective *N*-linked oligosaccharides of these enzymes in a two-step process. First, GNPT transfers a GlcNAc-1-phosphate residue from uridine diphosphate *N*-acetylglucosamine (UDP-GlcNAc) to the C6 position of a specific mannose residue in the high-mannose type oligosaccharides of the hydrolases. Later, the *N*-acetylglucosamine-1-phosphodiester α-*N*-acetylglucosaminidase (also known as “uncapping enzyme” or UCE) removes the terminal GlcNAc, exposing the M6P recognition signal. The mannose residues bearing the M6P marker are specifically recognized by mannose-6-phosphate receptors (M6PRs) located in the *trans*-Golgi network (TGN) (**Figure 1**). This recognition event plays a pivotal role in facilitating the efficient transport of hydrolases to lysosomes through the endosomal transport system. The acidic environment within endosomes triggers the separation between M6PRs and their substrates. Subsequently, M6PRs undergo recycling back to the TGN through the retromer system. As the endosomes undergo further acidification and maturation, lysosomal hydrolases transition into active mature enzymes and are conveyed into lysosomes (Braulke and Bonifacino, 2009).

Additionally, some soluble lysosomal membrane proteins, such as CLN3 and Niemann-Pick C1 (NPC1), are sorted into lysosomes via the M6P pathway, similar to lysosomal hydrolases (Pfeffer, 2019; Laqtom et al., 2022). Furthermore, extracellular lysosomal proteins can be internalized into the cytoplasm through the M6P-dependent endocytic pathway, which forms the basis of enzyme replacement therapy for lysosomal storage disorders (Banik et al., 2020).

Malfunctions in the M6P pathway can disrupt the transport of acid hydrolases to lysosomes, leading to lysosomal storage diseases (LSDs). These rare metabolic disorders result from the deficiency of specific lysosomal hydrolases. In LSD individuals, undegraded metabolites progressively accumulate within lysosomes, causing cellular abnormalities and impairments in various organs and systems throughout the body. The consequences of lysosomal storage diseases are often severe and can contribute to premature death (Platt, 2018).

### 2.1 The GlcNAc-1-phosphotransferase

GNPT plays a crucial role in initiating the formation of the M6P signal by introducing a phosphate group to mannose residues on lysosomal hydrolases. It functions as a heterodimeric complex with a molecular weight of 540 kDa, comprising three subunits (α, β, γ). The *GNPTAB* gene encodes the α and β subunits, which together produce a precursor protein weighing 190 kDa. This precursor encompasses the catalytic domain as well as structural modules responsible for the recognition and binding of lysosomal enzymes. The Golgi-resident site-1 protease (S1P) can cleave the peptide bond between Lys928 and Asp929 of the precursor, leading to its activation. On the other hand, the γ subunit is encoded by the *GNPTG* gene and generates a protein of approximately 97 kDa. The GNPTG protein contains a domain that specifically binds to mannose, thereby facilitating the recognition of substrates (Gorelik et al., 2022). Although GNPT was characterized nearly 40 years ago as pivotal in catalyzing M6P residue formation, its structural and functional characteristics remain subjects of ongoing investigation. Research to date indicates that GNPTAB and GNPTG cooperatively function in the M6P pathway, ensuring effective targeting of lysosomal enzymes. Activated GNPT selectively recognizes and binds UDP-GlcNAc and high-mannose-modified lysosomal enzymes. In this process, UDP-GlcNAc serves as the donor substrate for GNPT, tightly associating with the cavity of the GNPT catalytic center. The *N*-glycosylated lysosomal enzyme, as a receptor for GNPT, interacts with the flexible regions of GNPT Notch repeats and the DNA methyltransferase-associated protein (DMAP) interaction domain, binding to the active site. This binding locks GNPT into an active state, initiating the phosphorylation reaction. Through this reaction, GNPT transfers the GlcNAc-1-phosphate residue from UDP-GlcNAc to the C-6-hydroxyl position of the terminal or penultimate mannose, forming a polysaccharide containing GlcNAc-P-Man (Kudo et al., 2005; Kang et al., 2010; Gorelik et al., 2022; Li et al., 2022). Therefore, the knockout of GNPT results in the loss of M6P labeling and complete inactivation of lysosomal enzymes. The inactivated enzymes are then secreted extracellularly, leading to impaired degradation of autophagic and endocytic cargo and accumulation of undegraded materials within the lysosomal lumen (Braulke et al., 2023).

Mutations in the *GNPTAB* and *GNPTG* genes can result in the development of lysosomal storage disorders, specifically mucolipidosis type II (ML II, also known as I-cell disease) and mucolipidosis type III (ML III). Abnormalities in GNPT interfere with the regular transport of their substrates, lysosomal hydrolases, into the lysosomes. As a result, there is a significant buildup of degradation substrates, such as glycosaminoglycans (GAGs) and specific lipids, within the cells. Affected patients experience abnormal skeletal development, multiple osteoporosis, gingival hyperplasia, and a shortened lifespan. Furthermore, there is a notable increase in the levels of serum lysosomal hydrolases in these patients (Coutinho et al., 2012; Velho et al., 2015; Thelen and Zoncu, 2017).

### 2.2 The uncovering enzyme

*N*-acetylglucosamine-1-phosphodiester α-*N*-acetylglucosaminidase, commonly referred to as the uncovering enzyme (UCE), plays a crucial role in the M6P pathway by cleaving GlcNAc from GlcNAc-P-Man (Rohrer and Kornfeld, 2001). This cleavage event exposes the M6P recognition site of lysosomal hydrolases. UCE is a type I transmembrane protein comprising 515 amino acids and exists as a tetramer. It undergoes a dynamic cycle between the TGN and the plasma membrane (Rohrer and Kornfeld, 2001). The UCE protein is encoded by the *NAGPA* gene, which consists of 10 exons. Notably, UCE possesses six N-glycosylation sites and is initially synthesized as an inactive precursor. Subsequently, the TGN-resident Furin protease cleaves this precursor, converting it into its active form. UCE identifies GlcNAc and M6P residues on lysosomal hydrolases, cleaving the GlcNAc-P-mannose diester bond to yield mannose-6-P monester and produce free GlcNAc along with hydrolases modified with M6P (Gorelik et al., 2020). UCE exhibits strict substrate preferences, specifically hydrolyzing alpha-linked GlcNAc that is attached to a phosphate moiety, while other sugar modifications cannot fit precisely into its specialized GlcNAc-binding pocket. This attribute of UCE was elucidated through structural analysis by Gorelik et al., demonstrating that UCE recognizes the GlcNAc-P moiety on the polysaccharide substrate rather than the mannose portion (Gorelik et al., 2020).

The *UCE^-/-^* mice displayed normal viability, regular growth, and no observable histologic abnormalities. However, the plasma levels of six acid hydrolases were significantly elevated, ranging from 1.6- to 5.4-fold higher than the levels observed in wild-type mice. Significantly, the secreted hydrolases maintained GlcNAc-P-Man diesters, leading to a diminished binding affinity for the cation-independent mannose 6-phosphate receptor (CI-MPR), and they did not bind to the cation-dependent mannose 6-phosphate receptor (CD-MPR) at all (Boonen et al., 2009). The results obtained from this study suggest that even in the absence of UCE, there is still a level of weak binding between acid hydrolases and the CI-MPR. This residual binding is adequate to facilitate the sorting of acid hydrolases into lysosomes, effectively preventing the tissue abnormalities that typically arise in the absence of GNPT.

### 2.3 Mannose 6-phosphate receptors

After newly synthesized lysosomal hydrolases are tagged with M6P, they undergo recognition by the specific receptor known as M6PRs, facilitating their transport into lysosomes or secretion outside the cell (Griffiths et al., 1988; Dahms et al., 1989). There are two isoforms of M6PRs: CI-MPR, with a molecular weight of 300 kDa, and CD-MPR, with a molecular weight of 45 kDa. The former operates independently of divalent cations, while the latter necessitates their presence. Both CI-MPR and CD-MPR belong to the P-type lectin family as type I transmembrane glycoproteins (Dahms and Hancock, 2002). They exhibit binding affinity for specific oligosaccharides within the pH range of 6.5 to 6.7 and release those oligosaccharides at pH levels below 6. M6PRs are distributed across various cellular compartments, including the TGN, early endosomes (also known as sorting endosomes), recycling endosomes, late endosomes, and the plasma membrane. In order to prevent degradation by the acid hydrolases present in lysosomes, M6PRs actively avoid entering these organelles (Ghosh et al., 2003). Instead, they engage in regular cycling between these compartments, facilitated by sorting signals within their cytoplasmic domains, the adaptor protein complex (AP)-1, and Golgi-localized γ-ear-containing Arf-binding (GGA) proteins. The GGA proteins play a role in facilitating the back-and-forth movement between these organelles, while the return of M6PRs to the TGN is mediated by the retromer complex and Rab9 (Puertollano et al., 2001; Hirsch et al., 2003).

In addition to M6P, CI-MPR demonstrates binding affinity for a diverse range of ligands, encompassing M6P diester as well as non-glycosylated ligands like insulin-like growth factor II (IGF II) (Blanchard et al., 1998). This multifaceted binding capacity is why it is also commonly referred to as the IGF II receptor. The CI-MPR predominantly exists as a membrane dimer. Its extracellular region is composed of 15 homologous, 150-amino-acid repeat units, each belonging to the P-type carbohydrate recognition domain. Among them, domains(D) 3, 5, 9, and 15 specifically bind to M6P-labeled glycoproteins and are collectively referred to as M6P receptor homology (MRH) domains (Olson et al., 2014). In contrast, D11 specifically binds to IGF2. D1 and D2 work synergistically to stabilize the carbohydrate-binding loop of D3, resulting in a remarkable 1000-fold enhancement in its binding affinity to the M6P monoester. On the other hand, D5 demonstrates selective binding to the diester form of M6P (M6P-GlcNAc). Just like D3, the presence of neighboring domains enhances D5’s binding ability (Dwyer et al., 2020). D9 also displays a remarkable high affinity and selective binding to M6P monoesters, governed by a specific four-residue “QREY” binding motif comprising Q1283, R1325, E1345, and Y1351. Notably, this binding process does not require the cooperation of other structural domains of CI-MPR (Bochel et al., 2020). CD-MPR is significantly smaller than CI-MPR, consisting of just one P-type carbohydrate recognition domain at its extracellular end, enabling it to bind to a single M6P residue. Typically, CD-MPR exists in membranes predominantly as a dimer, although it can also be found as a monomer or trimer. The equilibrium between these various oligomeric forms is influenced by factors such as pH, temperature, and the presence of mannose 6-phosphate residues (Ghosh et al., 2003).

CD-MPR-deficient mice generally appear healthy, except for specific abnormalities related to the targeting of multiple lysosomal enzymes. These mice exhibit elevated blood levels of certain phosphorylated lysosomal enzymes and experience accumulation of undigested material within lysosomes (Ludwig et al., 1993). CI-MPR knockout mice have a survival period limited to day 15 of gestation, and their deaths primarily result from cardiac hyperplasia due to the inability to regulate free IGF-II levels (Sohar et al., 1998). However, simultaneous knockout of IGF-II prevents the death of these mice. Upon further examination of the embryos, a defect in lysosomal enzyme targeting and an elevation in phosphorylated lysosomal enzymes in amniotic fluid were observed. In the absence of CI-MPR, approximately 70% of the lysosomal enzymes were secreted, suggesting that CD-MPR could not fully compensate for the function of CI-MPR (Schellens et al., 2003). The findings from the knockout mice indicate that successful targeting of lysosomal enzymes requires both receptors, with each MPR possessing its own preferred subset of lysosomal hydrolases.

### 2.4 Lysosomal enzyme trafficking factor

LYSET (lysosomal enzyme trafficking factor), also known as TMEM251/GCAF, is a 20kD transmembrane protein primarily located within the Golgi apparatus and the cellular membrane (Braulke et al., 2023). Its encoding gene was discovered through whole-exome sequencing in two families affected by severe skeletal dysplasia. Notably, specific mutations within the TMEM251 gene—c.133C>T; p.(Arg45Trp) and c.215D>A; p.(Tyr72Ter)—were identified as crucial genetic triggers for this condition (Ain et al., 2021). Experimental validation via two distinct CRISPR whole-genome knockout-mediated loss-of-function screens confirmed TMEM251’s regulatory role in governing lysosomal biogenesis, prompting its renaming as LYSET. One screen aimed to identify factors associated with deficiencies in lysosomal degradation of RNF152, while the other utilized defects in cathepsin-mediated viral infections (including SARS-CoV-2) as criteria (Richards et al., 2022; Zhang et al., 2022a).

In TMEM251/LYSET knockout cells, approximately 40 lysosomal hydrolases were observed to be expelled into the extracellular environment instead of correctly localizing within lysosomes. This phenotype aligns with deficiencies resulting from *GNPT* and *CI-MPR* gene knockouts. Abnormal secretion of lysosomal enzymes in knockout cells led to compromised lysosomal function and disruption of lysosome-dependent pathways, such as membrane protein degradation, endocytosis, and autophagy (Pechincha et al., 2022).

*LYSET* gene knockout mice displayed hallmark features of ML II, characterized by elevated blood levels of lysosomal enzymes, enlarged lysosomes, and accumulation of electron-dense material within cells (Richards et al., 2022). To elucidate LYSET’s precise function within the M6P pathway, Zhang et al. conducted a comparative analysis involving knockouts of LYSET, GNPT, and CI-MPR. All knockout cells exhibited extracellular secretion of lysosomal hydrolases. Remarkably, in CI-MPR-depleted cells, the secreted hydrolases retained the M6P modification, indicating CI-MPR’s involvement solely in downstream recognition rather than upstream modification. Conversely, hydrolases secreted by cells with GNPT, LYSET single-knockout, and LYSET CI-MPR double-knockout lacked the M6P modification, indicating LYSET’s role in the early stages of the M6P pathway (Zhang et al., 2022a). At a molecular level, LYSET assumes a crucial role in coordinating the M6P pathway by reinforcing GNPTAB. LYSET co-localizes and interacts with GNPTAB within the Golgi apparatus. Its hydrophobic transmembrane domain plays a stabilizing role by supporting the hydrophilic transmembrane helices of the less stable GNPTAB α and β subunits. Consequently, LYSET can be considered a constituent of the GNPT complex responsible for anchoring and stabilizing the complex within the Golgi membrane (Pechincha et al., 2022). In the absence of LYSET, destabilization of the hydrophilic transmembrane domains within GNPT results in their incorrect relocation from the Golgi complex to the lysosomes. Consequently, proteins modified with M6P, including lysosomal enzymes, undergo substantial reduction due to this mis-localization (Pechincha et al., 2022; Richards et al., 2022). However, it remains unclear whether LYSET consistently binds to GNPT, engages with other Golgi proteins, and the underlying reasons for the GNPT complex’s reliance on LYSET for stability and retention within the Golgi membrane await clarification.

## 3 M6P pathway and viral infection

### 3.1 The M6P-dependent cathepsins and viral infection

Cathepsins derive their name from the Greek word “kathepsein,” meaning “to digest,” indicating their role as proteases active within a slightly acidic environment. Over time, the term has evolved to encompass acidic proteases found within lysosomes. Currently, 15 cathepsins have been identified in the human body and are classified into distinct classes based on their active site residues, namely serine, aspartate, and cysteine proteases (McGrath, 1999).

Similar to other lysosomal hydrolases, nascent cathepsins are folded and initially glycosylated in the ER before undergoing transportation to the Golgi for further processing. Cathepsins in the Golgi also necessitate M6P-labeling for appropriate sorting into the endosomal/lysosomal system, facilitated by the M6P pathway. Within the acidic environment of lysosomes, cathepsin precursors undergo hydrolysis, shedding their pre-structural domains to become active proteases (Reiser et al., 2010).

The intricate relationship between the M6P pathway and viral infection is highlighted by the multifaceted involvement of cathepsins in the host infection process of specific viruses. Cathepsins play pivotal roles across various stages of host cell infection, including viral entry, replication, release, spreading, and evasion from host immune responses. Elaborate processes and molecular mechanisms concerning cathepsin involvement in distinct viral infections have been extensively reviewed elsewhere and will not be reiterated here (Scarcella et al., 2022).

**Figure 2:**
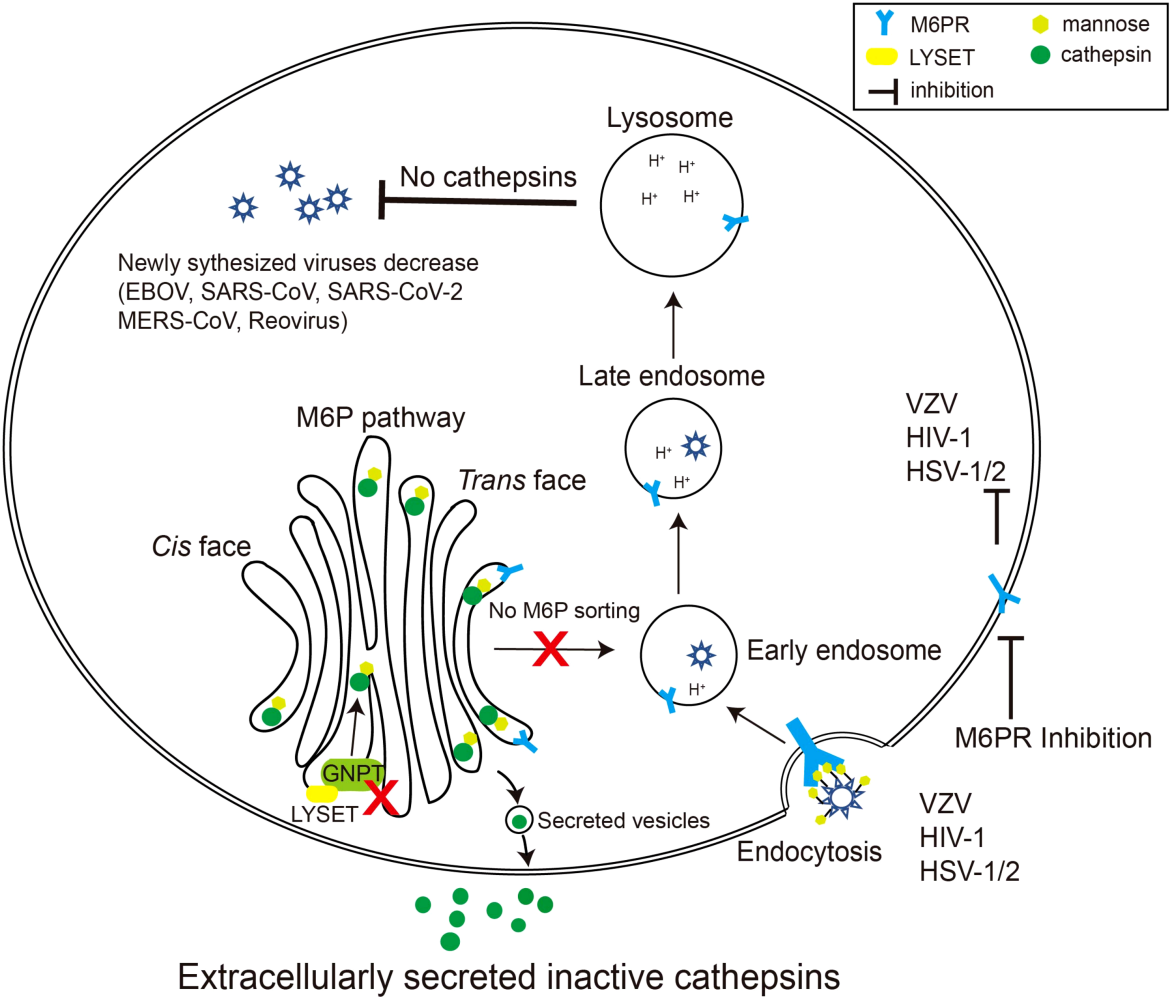
Schematic diagram of viruses exploiting the M6P pathway. Certain species of viruses, such as reovirus, EBOV, and coronavirus, require cathepsins, the final product of the M6P pathway, to modify their surface glycoproteins. When LYSET and GNPT are deficient, cathepsins are inactive and secreted out of the cell. Therefore, the surface proteins of the viruses cannot be modified, resulting in a decrease in the production of the virus in the host. Other viruses including varicella zoster virus (VZV), HSV-1/2, and HIV-1 can use mannose or M6P residues on their envelope glycoproteins to bind M6PRs, thereby triggering the endocytosis process of the viruses. Inhibiting the activity of M6PRs will inhibit the infection by these viruses.

Given the critical role of the M6P signal in cathepsin maturation and activity, coupled with the substantial contribution of cathepsins to specific virus invasions, it is evident that the M6P signal constitutes a crucial component in the viral infection process (**Figure 2**).

### 3.2 M6P regulators and viral infection

Viruses initiate infection by engaging cell surface receptors, triggering endocytosis. Subsequently, within the endolysosomal system, they leverage environmental cues to facilitate the transfer of their genetic material across the cell membrane into the cytoplasm. During late-stage endocytosis, viruses co-opt vital lysosomal functions to aid their escape and prevent degradation (Marsh and Helenius, 2006; Helenius, 2018; Braulke et al., 2023). The unexpected revelation that lysosomal membrane proteins NPC1 and LAMP1 act as receptors for Ebola and Lassa virus glycoproteins, respectively, diverges from the conventional viral receptor binding pattern at the cell surface (Carette et al., 2011; Miller et al., 2012; Cohen-Dvashi et al., 2015; Qian et al., 2020; Zhang et al., 2022b). These discoveries underscore the viral capacity to shift from plasma membrane binding to intracellular receptor interactions. Recent investigation has unveiled the lysosomal transmembrane protein TMEM106B as an intracellular receptor for SARS-CoV-2 (Baggen et al., 2023). Viruses might delay the exposure of receptor binding domains until they reach the inaccessible endolysosomal system, evading the host’s humoral immune response (Miller et al., 2012). Additionally, viruses triggering cellular entry through lysosomal protease cleavage of structural proteins are vulnerable to disruptions in components of the M6P-mediated lysosomal transport pathway.

The initial evidence of GNPT’s significance in viral infection arose from a genome-wide haploid genetic screen in human cells, targeting host factors crucial for Ebola virus (EBOV) entry. Among 67 loss-of-function mutants detected, a noteworthy subset of 6 mutants was associated with the GNPT subunit GNPTAB (Carette et al., 2011). Similarly, Snyder et al. conducted a whole-genome knockout screening using CRISPR-Cas9, identifying GNPTAB as one of seven essential genes for triggering cell death in mouse embryonic fibroblasts following reovirus infection (Snyder et al., 2022). Despite these screenings revealing GNPT’s involvement in specific viral infection processes, GNPT remained secondary in focus. Subsequent investigations into its precise role in these viral infections were not pursued in either study.

Flint et al. conducted another CRISPR genome-wide knockout screen, confirming the pivotal role of GNPTAB in EBOV infection, which consequently triggers cell death in Huh7 cells (human hepatocarcinoma cells) (Flint et al., 2019). Subsequent experiments have shown that the deletion of GNPTAB in HAP1 cells (human near-haploid chronic myelogenous leukemia) hindered EBOV infectivity. However, restoring GNPTAB expression reversed this impediment. Additionally, fibroblasts from individuals affected by ML II, a condition associated with GNPTAB gene mutations, demonstrate resistance to the EBOV, while cells from their unaffected parents facilitate infection. The knockout of GNAPTAB in HAP cells is strongly associated with the diminished activities of cathepsin B and L, both crucial for EBOV entry. However, the exact involvement of GNPTAB in EBOV infection by regulating cathepsin maturation is still under investigation. This uncertainty arises because *CTSB* and *CTSL* (the genes encoding cathepsin B and cathepsin L, respectively) were identified as non-significant hits in Flint’s original screen (Flint et al., 2019). PF-429242, an inhibitor targeting SKI-1/S1P protease necessary for processing GNPTAB precursor to activate GlcNAc-phosphotransferase activity, exhibits significant efficacy in inhibiting EBOV infection. This finding underscores the potential of GNPTAB as a promising pharmaceutical target against EBOV infection. However, considering the linkage of GNPTAB defects with lysosomal storage disorders, its activity should be cautiously and temporarily modulated to avert severe lysosomal storage disease symptoms. Flint’s research demonstrates that despite residual GNPT activity observed in cells from individuals displaying milder clinical symptoms of ML III associated with GNPT defects, the replication capacity of EBOV is considerably diminished (Flint et al., 2019). This supports the notion that partial inhibition of GNPTAB effectively thwarts EBOV invasion. Recently, two independent groups have successfully elucidated the crystal structures of both the catalytic subunit GNPTAB and the regulatory subunit GNPTG within GNPT (Gorelik et al., 2022; Li et al., 2022). These findings have provided invaluable insights for the development of drugs aimed at precisely modulating GNPT activity to counter EBOV.

Concurrently with Snyder’s study, Richards et al. conducted an independent and comparable genome-scale CRISPR-Cas9 screen to identify crucial host genes involved in reovirus infection (Richards et al., 2022). While Snyder’s study used MEFs and a mouse CRISPR knockout library, Richards employed human glioblastoma cells (U87MG) and a human-based Brunello library. Both screens identified GNPTAB and CTSL, as recognized participants in the M6P pathway. However, Richards’ investigation uniquely revealed LYSET (TMEM251), a previously uncharacterized gene absent in Snyder’s study. The deletion of LYSET in U87MG and 293FT cells provided significant protection against cell death subsequent to reovirus infection, mirroring the impact seen with GNPTAB or site-1 protease (S1P) knockout. Additionally, LYSET deletion impeded the entry of other cathepsin-dependent viruses, encompassing EBOV and the Omicron strain of SARS-CoV-2, in diverse cell lines (**Figure 2**). In cells expressing TMPRSS2, the SARS-CoV-2 Delta variant primarily utilizes the serine protease TMPRSS2 for entry, but in cells with very low TMPRSS2 expression, it resorts to cathepsin for entry. Knockout of LYSET in TMPRSS2-deficient cells blocked the entry of the SARS-CoV-2 Delta variant, but this effect was not observed in cells with TMPRSS2 expression. This demonstrates that LYSET plays a role in viral invasion by modulating the proper sorting of cathepsins.

### 3.3 M6P receptors and viral infection

The surface proteins of certain viruses are modified by M6P, enabling their interaction with CD-MPR and CI-MPR, thereby becoming ligands for M6PRs. Consequently, these viruses exploit the M6PRs located on the cell surface as an entry receptor or co-receptor for invading host cells. The selection of a specific receptor varies among distinct viruses or different subtypes of the same virus. These viruses encompass *Herpesviridae* (HSV*-*1, HSV*-*2), varicella zoster virus (VZV) and human immunodeficiency virus 1 (HIV-1) among others (**Figure 2**) (Brunetti et al., 1994; Mbemba et al., 1994; Brunetti et al., 1995; Chen et al., 2004; Dohgu et al., 2012; Díaz-Salinas et al., 2018; Ohka et al., 2022). **Table 1** enumerates the specific surface glycoprotein ligands for each virus and their corresponding host M6PR entry receptors (**Table 1**). Genetically disrupting the host cell’s M6PRs or competitively binding M6PRs with exogenous mannose-6-phosphate substantially impedes the dissemination of VZV and HSV-1/2, and HIV-1, indicating the potential of M6P or M6P analogs as therapeutic avenues for managing viral infections (Brunetti et al., 1994; Brunetti et al., 1995; Chen et al., 2004; Dohgu et al., 2012). Unlike the M6PRs present on the cell surface, the intracellular M6PRs play a role in transporting internalized viral particles to acidic endosomes for uncoating and the subsequent release of the viral genome. Enterovirus 71 (EV71), responsible for causing hand-foot-mouth disease, relies on CI-MPR present on the host cell surface as a co-receptor along with scavenger receptor B2 (SCARB2) to facilitate its entry into the cell. Once the virus is internalized, it is directed to the late endosome for uncoating, a process in which CD-MPR is essential (Ohka et al., 2022). However, the precise role of CD-MPR in either transporting EV71 particles into the late endosome or delivering cathepsins to the late endosome to facilitate the final uncoating phase has yet to be investigated.

**Table 1:** Viral surface glycoproteins and their corresponding host M6PRs.

Viruses	Viral glycoproteins	Experimental models	M6PR functions	Reference(s)
VZV	gE and gI	Human melanoma MeWo cells and epidermal cells from VZV-infected patients	Intracellular CI-MPRs divert newly enveloped VZV to late endosomes, and plasmalemmal CI-MPRs are necessary for entry by cell-free VZV	(Chen et al., 2004)
HSV-1/2	gD	Monkey Vero cells, human and mouse fibroblasts, human epithelial tonsil cells, 293, CHO, and human R970 cells	M6PRs bind to viral gD and act as cellular receptors for HSV-1/2	(Brunetti et al., 1994; Brunetti et al., 1995)
HIV-1	gp120	Human and murine microglia, primary culture of mouse brain microvascular endothelial cells (BMECs), and CI-MPR knockout mice	CI-MPRs are highly expressed in microglial nodules in human brains with HIV encephalitis, acting as an important receptor used by HIV-1 to replicate and cross the BBB.	(Mbemba et al., 1994; Suh et al., 2010; Dohgu et al., 2012)
Rotavirus	ND	Monkey epithelial cell line MA104, the murine fibroblast L929 cell line, and human intestinal epithelial Caco-2 cells	Most rotavirus strains require M6PRs to infect efficiently	(Díaz-Salinas et al., 2014; Díaz-Salinas et al., 2018)
EV71	ND	Human rhabdomyosarcoma RD cells and monkey Vero cells	CD-MPR is required for uncoating EV71 in mature late endosomes	(Ohka et al., 2022)

* ND, not determined.

The intracellular M6PRs might play a role in transporting newly assembled viruses within host cells. VZV is the causative agent of chickenpox and, despite its high infectivity, exhibits slow dissemination within infected individuals. In VZV-infected *in vitro* cultured cells like MeWo cells, the release of infectious virions into the extracellular space is hindered. Virus transmission between cells relies on a gradual cell-to-cell contact mechanism, possibly involving fusion between infected and neighboring cells (Zerboni et al., 2014). It’s postulated that the disparity in transmission rates of VZV between hosts and within hosts is due to its transmission through free infectious virions among hosts and through cell-to-cell contact during intercellular transmission (Gershon and Gershon, 2010). Detailed examination indicates that VZV acquires an envelope within specific areas of the TGN, where lipid components and M6P-modified envelope glycoproteins are present. Concurrently, traffic vesicles containing CI-MPR are formed, aiding in the transport of newly packaged virus particles to the late endosome by binding to M6P-modified glycoproteins. Within the acidic environment of the late endosome, the virus undergoes degradation before being released via exocytosis, leading to the presence of noninfectious, anucleate, and polymorphic enveloped particles in the extracellular space. In M6PR-deleted MeWo cells, the transportation of VZV to late endosomes ceases, allowing the virus to bypass degradation and instead be directly secreted into extracellular vesicles while retaining its infectiousness. Biopsy analysis of VZV-infected human skin suggests that decreased CI-M6PR expression in maturing superficial epidermal cells prevents the redirection of VZV to endosomes. Consequently, these cells continuously release infectious VZV particles (Chen et al., 2004).

## 4 Conclusions

Some viruses such as EBOV, coronaviruses, and reovirus heavily rely on key members of the host’s M6P pathway, particularly GNPTAB, and LYSET. The absence of GNPTAB and LYSET significantly affects these viruses’ ability to enter cells (Richards et al., 2022). This reliance is due to their requirement for cathepsins, products of the M6P pathway, to modify their surface proteins. Apart from these viruses, many others that rely on cathepsins for invasion include feline morbillivirus (FeMV), Nipah virus (NiV), porcine reproductive and respiratory syndrome virus (PRRSV), respiratory syncytial virus (RSV), among others. Their relationship with the M6P pathway requires further investigation.

Moreover, certain viruses like HIV-1 ingeniously exploit M6PRs for invasion and infection (Dohgu et al., 2012). However, viruses using M6PRs often possess alternative, more generalized mechanisms to invade cells through cell surface receptors triggering endocytosis. The relationship between these viruses and M6PR needs specific experimental conditions for exploration.

The identification of LYSET as a new regulatory member of the M6P pathway implies that mysteries surrounding this pathway persist. A deeper exploration of the M6P pathway not only lays new foundations for understanding cellular biosynthesis but also offers fresh perspectives on virus-host interactions.

In recent years, advancements in structural biology techniques have continuously unveiled the three-dimensional structures of M6P pathway components and viral proteins. This progress will aid in targeting relevant proteins. Screening and identifying small molecules targeting these proteins to modulate the M6P pathway represent a novel antiviral strategy.

## Author contributions

QL: Writing – original draft, Investigation, Visualization. WW: Data curation, Investigation, Methodology, Writing – original draft. LX: Data curation, Investigation, Methodology, Writing – original draft. QZ: Conceptualization, Writing – review & editing. HW: Conceptualization, Writing – original draft.
